# Remarks on Medical Organization and Reform, French and English

**Published:** 1846-07

**Authors:** 


### Remarks on Medical Organization and Reform, French and English. By *Edwin Lee.* 8vo, pp. 121. London, 1846.

In our own struggle for medical reform, Dr. Lee believes it may be useful to exhibit the systems of medical organization which prevail in France, Germany and Italy. These, however, are for the most part far too elaborate, methodical, and despotic, to admit of being transplanted among us. Indeed, before admiring and wishing to imitate them, we should observe how indifferently they work in their own respective countries, among persons much more accustomed to submissiveness to authority than we are; and do we not find that the profession is everywhere in a very discontented and unsatisfactory condition? That this is the case in France is notorious, and much that Mr. Lee mentions as regards that country is rather what the Congress desires, that what really exists; and in respect to essentials, we do not hesitate to affirm that, as regards the mass of medical men, their condition is much more to be deplored than in our own. We are not advocates for the transplantation of any cut-and-dried system from one country to another, in which the manners and institutions are not fitted for its reception. Each country has its peculiar abuses to remove or diminish; for, after all, the great thing we want is to be allowed unrestricted means of improving our professional position. This can only be done by improving our education, by bringing us more together, so that we may discern and support our true interests, and by preserving us and the public, from the injurious competition of uneducated and ignorant persons. The two existing medical incorporations are quite competent to these ends, but are unwilling to accomplish them. It behoves, then, the *great mass of practitioners to unite boldly and firmly together.* Their forbearance has hitherto been met with contumely, neglect, and opposition. They have the power in their own hands, either of bringing their adversaries (for we regret the conduct of the corporate bodies justifies this term), to a capitulation, or of erecting themselves into so important a rival institution as to render this a matter of no consequence. Their organization is the first essential object. That effected, they may do all they can legitimately desire; without it, they will continue to be tantalized, derided, and deceived as they have hitherto been.

Much of Mr. Lee's work is taken up with an exposition of the abuses prevailing in the Colleges of Physicians and Surgeons; but we think the profession is by this time fully aware of the nature and extent of these, as also of the impossibility of their amendment by any means short of those we have adverted to.

